# Muscarinic Toxin 7 Signals Via Ca^2+^/Calmodulin-Dependent Protein Kinase Kinase β to Augment Mitochondrial Function and Prevent Neurodegeneration

**DOI:** 10.1007/s12035-020-01900-x

**Published:** 2020-03-20

**Authors:** Ali Saleh, Mohammad Golam Sabbir, Mohamad-Reza Aghanoori, Darrell R. Smith, Subir K. Roy Chowdhury, Lori Tessler, Jennifer Brown, Eva Gedarevich, Markos Z. Kassahun, Katie Frizzi, Nigel A. Calcutt, Paul Fernyhough

**Affiliations:** 1 Division of Neurodegenerative Disorders, St Boniface Hospital Albrechtsen Research Centre, R4046 - 351 Taché Ave, Winnipeg, Manitoba R2H 2A6 Canada; 2 Department of Pharmacology and Therapeutics, University of Manitoba, Winnipeg, MB Canada; 3 Department of Pathology, University of California San Diego, La Jolla, CA USA

**Keywords:** Antimuscarinic, Bioenergetics, Diabetic neuropathy, CIPN, Mitochondria, Nerve regeneration

## Abstract

Mitochondrial dysfunction is implicated in a variety of neurodegenerative diseases of the nervous system. Peroxisome proliferator–activated receptor-γ coactivator-1α (PGC-1α) is a regulator of mitochondrial function in multiple cell types. In sensory neurons, AMP-activated protein kinase (AMPK) augments PGC-1α activity and this pathway is depressed in diabetes leading to mitochondrial dysfunction and neurodegeneration. Antimuscarinic drugs targeting the muscarinic acetylcholine type 1 receptor (M_1_R) prevent/reverse neurodegeneration by inducing nerve regeneration in rodent models of diabetes and chemotherapy-induced peripheral neuropathy (CIPN). Ca^2+^/calmodulin-dependent protein kinase kinase β (CaMKKβ) is an upstream regulator of AMPK activity. We *hypothesized* that antimuscarinic drugs modulate CaMKKβ to enhance activity of AMPK, and PGC-1α, increase mitochondrial function and thus protect from neurodegeneration. We used the specific M_1_R antagonist muscarinic toxin 7 (MT7) to manipulate muscarinic signaling in the dorsal root ganglia (DRG) neurons of normal rats or rats with streptozotocin-induced diabetes. DRG neurons treated with MT7 (100 nM) or a selective muscarinic antagonist, pirenzepine (1 μM), for 24 h showed increased neurite outgrowth that was blocked by the CaMKK inhibitor STO-609 (1 μM) or short hairpin RNA to CaMKKβ. MT7 enhanced AMPK phosphorylation which was blocked by STO-609 (1 μM). PGC-1α reporter activity was augmented up to 2-fold (*p* < 0.05) by MT7 and blocked by STO-609. Mitochondrial maximal respiration and spare respiratory capacity were elevated after 3 h of exposure to MT7 (*p* < 0.05). Diabetes and CIPN induced a significant (*p* < 0.05) decrease in corneal nerve density which was corrected by topical delivery of MT7. We reveal a novel M_1_R-modulated, CaMKKβ-dependent pathway in neurons that represents a therapeutic target to enhance nerve repair in two of the most common forms of peripheral neuropathy.

## Background

The field of innervation of intraepidermal nerve fibers (IENFs) within the skin is plastic and maintained through a combination of collateral sprouting and regeneration that is regulated partly by neurotrophic factors [1, 2]. Distal dying-back of nerve fibers is observed in many peripheral neuropathies including those associated with diabetes, chemotherapy-induced peripheral neuropathy (CIPN), Friedreich’s ataxia, Charcot-Marie-Tooth disease type 2, and human immunodeficiency virus (HIV). There are no therapies for any of these neuropathies. Intriguingly, all of these diseases display some degree of mitochondrial dysfunction [3, 4].

The true prevalence of diabetic sensory neuropathy is not known and reports vary in their estimates from 10 to 90% in diabetic patients, depending on the criteria and methods used to define neuropathy [5]. The human and economic burden of diabetic neuropathy and its consequences in the form of painful neuropathy, foot ulceration, and amputation are considerable for both patients and healthcare systems [6]. The most common form of diabetic neuropathy in type 1 and type 2 patients, symmetrical sensorimotor polyneuropathy, exhibits pathological changes in both large and small fibers of peripheral nerves. Some of the earliest pathological features include the appearance of swellings in unmyelinated fibers that project into the epidermis, and there is dying-back of these distal axons leading to reduced IENF density in skin [5, 7–10]. Further, defective axon sprouting and regeneration impedes tissue re-innervation [11]. These indices of nerve pathology are also observed in rodent models of both type 1 and type 2 diabetes [12, 13], allowing for investigation of both putative pathogenic mechanisms and potential therapies.

Depending on the specific drug and dose regimen, CIPN can afflict between 20 and 85% of cancer patients receiving these drugs at standard doses and nearly 100% of patients at higher doses. Many patients with CIPN experience neuropathic pain, presenting as allodynia, hyperalgesia, and spontaneous pain. CIPN may resolve with cessation of chemotherapy, but in 30–70% of cases, it persists for months to years after treatment has concluded, or even increases in severity [14]. Patients with CIPN develop distal dying back of nerve fibers leading to loss of IENF, and this pathology is also seen in rodent models of CIPN [3].

Retraction or degeneration of IENF has been suggested to be the result of impaired mitochondrial function and local energy production. Growth cone motility is required to maintain fields of innervation and consumes 50% of ATP supplies in neurons due to high rates of local actin treadmilling [15]. In particular, unmyelinated axons are more energetically demanding than myelinated axons, consuming 2.5–10-fold more energy per action potential [16]. Mitochondria are known to concentrate in regions of high metabolic demand [17, 18], and sensory terminal boutons are packed with mitochondria [19, 20]. Our work in rodent models of type 1 and 2 diabetes has demonstrated that hyperglycemia triggers nutrient excess in neurons that, in turn, mediates a phenotypic change in mitochondria through alteration of the AMP-activated protein kinase (AMPK)/peroxisome proliferator–activated receptor γ coactivator-1α (PGC-1α) signaling axis [21]. A well-characterized upstream activator of AMPK is Ca^2+^/calmodulin-dependent protein kinase kinase β (CaMKKβ) which directly phosphorylates AMPK at its activation domain [22, 23] and forms a complex with AMPK [24]. This vital energy-sensing metabolic pathway modulates mitochondrial function, biogenesis, and regeneration [25–27]. The bioenergetic phenotype of mitochondria in diabetic neurons is characterized by inner membrane depolarization, reduced expression of respiratory chain components [28, 29], and suboptimal spare respiratory capacity [29–31], without remarkable ultrastructural alterations [32, 33].

Studies in peripheral nervous system (PNS) neurons demonstrate that acetylcholine (ACh) signaling through muscarinic receptors acts as a regulator of electrical activity and axonal growth cone motility during development. In sympathetic neurons, ACh activation of muscarinic acetylcholine type 1 receptor (M_1_R) mobilizes internal Ca^2+^ stores leading to closure of M-type K^+^ channels (Kv7 subtypes) and an enhancement of slow depolarization and discharge [34]. In embryonic neurons, ACh can modulate axonal growth in a positive or negative manner based upon context [35–37]. Furthermore, both adult sensory ganglia and the target of sensory neurons, keratinocytes within the epidermis, synthesize and secrete ACh [38, 39]. Adult rat sensory neurons of the dorsal root ganglia (DRG) express a peripheral form of choline acetyl transferase (pChAT), exhibit ChAT activity, have low AChE activity, and express multiple muscarinic receptors including M_1_R [38, 40, 41]. It is therefore becoming increasingly recognized that adult sensory neurons are cholinergic and amenable to manipulation by drugs that modulate cholinergic systems.

We have recently shown that selective or specific antagonists of M_1_R, including pirenzepine and muscarinic toxin 7 (MT7), induce a dose-dependent elevation of neurite outgrowth in adult sensory neurons [42–44]. Importantly, these drugs were also able to afford protection against several different forms of peripheral neuropathy. The exact mechanism of M_1_R antagonist–driven neurite outgrowth and nerve repair is, as yet, poorly described. To advance understanding of the downstream consequences of M_1_R antagonism, we tested the hypothesis that MT7, the only specific M_1_R antagonist (or allosteric modulator) [45], enhances mitochondrial function to drive neurite outgrowth via activation of the CaMKKβ/AMPK signal transduction pathway. Our attention was drawn to this possibility by the recent report that polymorphisms in the CaMKKβ gene are associated with susceptibility to HIV neuropathy [46]. To test our hypothesis, we performed mechanistic studies in vitro using adult sensory neuron cultures and assessed the capacity of MT7 to prevent neurodegeneration and drive nerve fiber repair in animal models of type 1 diabetes and CIPN.

## Methods

### Adult Rat DRG Sensory Neuron Culture

Animal protocols carefully followed the Canadian Committee on Animal Care (CCAC) guidelines. Adult male Sprague Dawley rats, approximately 5–8 weeks old and weighing 275–325 g, were fasted overnight then made diabetic with a single i.p. injection of 85 mg/kg streptozotocin (STZ) (Sigma-Aldrich, Oakville, ON, Canada) which causes degeneration of beta cells within the islets of Langerhans of the pancreas and induces experimental type 1 diabetes mellitus. Within weeks of induction, STZ-diabetic rats develop deficits in nerve conduction velocity and changes in pain perception threshold that reflect the clinical condition [47] and neuropathy in this model is not a consequence of direct STZ-induced neurotoxicity [48]. We confirmed the onset of sensory neuropathy using paw withdrawal time to a radiant heat stimulus [49], and only STZ-induced diabetic rats with blood glucose levels > 19 mM were selected for dissection and cell culture. Sensory neurons were isolated from the DRG and dissociated using previously described methods [42]. DRG neurons isolated from ganglia from all levels from these rats were plated onto poly-d-l-ornithine hydrobromide and laminin-coated multi-well plates for neuronal survival and axon outgrowth studies and 25 mm glass cover slips for immunocytochemistry. Neurons were grown in defined Hams F-12 medium (Life Technologies, Burlington, ON, Canada) with modified Bottenstein and Sato’s N2 medium (with no insulin) containing 0.1 mg/ml transferrin, 20 nM progesterone, 100 μM putrescine, 30 nM sodium selenite, and 1 mg/ml BSA, and supplemented with the following neurotrophic factors (NTFs): 0.1 ng/ml nerve growth factor (NGF), 0.1 ng/ml neurotrophin-3 (NT-3), and 1 ng/ml glial cell line–derived neurotrophic factor (GDNF) (all obtained from Sigma-Aldrich, Oakville, ON, Canada). Neurons derived from control animals were exposed to 10 mM glucose and 0.1 nM insulin. Neurons from STZ-induced diabetic rats were cultured in the presence of 25 mM glucose without adding insulin to mimic physiological conditions of diabetes in rats. Cultures were treated with MT7 (Alomone Labs, Jerusalem, Israel), STO-609 (Tocris, Minneapolis, MN, USA), or pirenzepine (Sigma-Aldrich, Oakville, ON, Canada).

### Assessment of Total Neurite Outgrowth

Rat neurons grown on glass cover slips were imaged directly for GFP fluorescence or fixed with 4% paraformaldehyde in phosphate-buffered saline (PBS, pH 7.4) for 15 min at room temperature and permeabilized with 0.3% Triton X-100 in PBS for 5 min. Cells were then incubated in blocking buffer (Roche, Indianapolis, IN, USA) diluted with FBS and 1.0 mM PBS (1:1:3) for 1 h then rinsed three times with PBS. The primary antibody used was against β-tubulin isotype III, which is neuron-specific (1:1000; Sigma-Aldrich, Oakville, ON, Canada). For CaMKKβ immunocytochemistry, we used antibody at a dilution of 1:1000 (Santa Cruz Biotechnology, Dallas, TX, USA). The antibody was added to all wells, and plates were incubated at 4 °C overnight. The following day, the coverslips were incubated with CY3-conjugated secondary antibodies (Jackson ImmunoResearch Laboratories, West Grove, PA, USA) for 1 h at room temperature and then mounted and imaged using a Carl Zeiss Axioscope-2 fluorescence microscope equipped with an AxioCam camera. Images were captured using AxioVision4.8 software. For GFP imaging, we used a Carl Zeiss LSM510 confocal microscope. Quantification of total neurite outgrowth was performed by measuring the mean pixel area of captured images using ImageJ software (adjusted for the cell body signal). All values were adjusted for neuronal number post acquisition [42]. In this culture system, the level of total neurite outgrowth has been validated to be directly related to an arborizing form of axonal plasticity and homologous to collateral sprouting in vivo [50].

### Luciferase Reporter Constructs for PGC-1α, Mutant Construct for CaMKKβ, and Cell Transfection of Adult Sensory Neurons

Reporter plasmid with the wild-type PGC-1α promoter upstream from luciferase was kindly donated by Dr. Michael Czubryt (University of Manitoba). DRG neurons (30 × 10^3^) were transfected in triplicate with 1.8 μg of PGC-1α promoter/*luciferase* plasmid DNA and 0.2 μg of pCMV-*Renilla* (Promega, Madison, WI, USA) using the Amaxa electroporation kit for low numbers of cells according to the manufacturer’s instructions (ESBE Scientific, Toronto, ON, Canada). Neurons were lysed using passive lysis buffer provided with the Dual-Luciferase Reporter Assay System (Promega, Madison, WI, USA). The luciferase activity was measured using a luminometer (model LMAXII; Molecular Devices, Sunnyvale, CA, USA). Twenty microliters of each sample was loaded in a 96-well plate and was mixed with 100 μl of Luciferase Assay Reagent II, and firefly luciferase activity was first recorded. Then, 100 μl of Stop-and-Glo Reagent was added, and Renilla luciferase activity was measured. All values are normalized to Renilla luciferase activity. Plasmid over-expressing a wild type or mutant of CaMKKβ was also transfected into adult neurons using the Amaxa machine as described above. The mutant construct, comprised of multisite mutations at S129D, S133D, and S137D, and a wild-type construct were kindly donated by Dr. Anthony Means (Duke University Medical Center) as described [51]. The mutant construct exhibited mutations at S129, S133, and S137 leading to reduced phosphorylation of the enzyme that inhibited the autonomous activity of the enzyme.

### Short Hairpin RNA–Based Blockade of CaMKKβ Signaling in DRG Neurons

For short hairpin RNA (shRNA)–based gene silencing, a GFP-expressing clone specific for CaMKKβ (clone ID: V3LMM_453709) was obtained from Open Biosystems (Lafayette, CO, USA). The pGIPz lentiviral system for mice and humans from the Open Biosystems database is held at the University of Manitoba, Winnipeg, Canada. A control scrambled shRNA unrelated to the CaMKKβ sequence was used as a negative control for lentiviral transduction [52]. For shRNA-based gene silencing studies, DRG neurons were transduced at 20× infectious unit in the presence of polybrene (8 μg/ml) for 2 h at 37 °C, complete medium was added, and neurons were cultured for additional 48 h. Fluorescence images derived from GFP-expressing virally transduced neurons were acquired by using a LSM510 confocal microscope (Carl Zeiss) with × 20 air objective. For dual transfection, neurons were initially transfected with plasmid for PGC-1α and, then after 48 h, were transduced with lentivirus carrying shRNA to CaMKKβ.

### SiRNA-Based Silencing of CaMKKβ in Neurons

The lipid nanoparticle (LNP)-based small interfering RNA (siRNA)-mediated knockdown of CaMKKβ was achieved as described previously [53]. Briefly, the cells were transfected with a cocktail of 3 siRNAs designed against a variety of sites within the gene (see [53] for siRNA sequence data). The siRNAs were encapsulated in LNPs as described previously [54]. The siRNA-LNPs were prepared using a microfluidic micromixer by Precision NanoSystems Inc., Vancouver, BC, Canada. For the encapsulation, the desired amount of siRNAs (0.056 μg siRNA/μmol of lipid) was dissolved in the formulation buffer (25 mmol/l sodium acetate, pH 4.0). The siRNA/LNP preparation was added to the cultured cells where transfection reached 80–90% efficiency with no toxicity, and knockdown was achieved within 2 days in adult sensory neurons.

### Western Blotting for CaMKKβ, P-AMPK, and Other Proteins

Rat DRG were harvested after treatments in ice-cold stabilization buffer containing 0.1 M PIPES (pH 6.9), 5 mM MgCl_2_, 5 mM EGTA, 0.5% Triton X-100, 20% glycerol, 10 mM NaF, 1 mM PMSF, and protease inhibitor cocktail. Mouse trigeminal ganglia were rapidly dissected, snap-frozen in liquid nitrogen, and stored at − 70 °C until assay. Protein assay was performed using the DC protein assay (Bio-Rad, Hercules, CA, USA), and Western blot analysis was conducted. The samples (5 μg total protein/lane) were resolved on a 10% SDS-PAGE gel and electroblotted (100 V, 1 h) onto a nitrocellulose membrane. Blots were then blocked in 5% non-fat milk (containing 0.05% Tween) overnight at 4 °C, rinsed in TBS-T, and then incubated with the primary antibodies to the following proteins: CaMKKβ (1:1000, Santa Cruz Biotechnology), CaMKKα (1:1000, Santa Cruz Biotechnology), phosphorylated CaMK1 (Thr-177, P-CaMK1; 1:1000; LifeSpan BioSciences, Seattle, WA, USA), phosphorylated CaMKIV (Thr-196 and Thr-200, P-CaMKIV; 1:750; Abcam, Cambridge, MA, USA), phosphorylated AMPK (P-AMPK on Thr-172; 1:500; Santa Cruz Biotechnology Inc., Santa Cruz, CA, USA; Cell Signaling Technology, Boston, MA, USA), total AMPK (T-AMPK; 1:500, Santa Cruz Biotechnology), PGC-1α (1:500, Santa Cruz Biotechnology), and phosphorylated acetyl coenzyme A carboxylase (P-ACC; 1:2000; Abcam, Cambridge, MA, USA). Total extracellular–regulated protein kinase (T-ERK; 1:2000, Santa Cruz Biotechnology) and/or actin were probed as loading controls. Previous studies have shown that the expression of T-ERK does not change in intact DRG or cultures from diabetic rats [55]. Secondary antibody was applied for 1 h at room temperature after 5–6 washes of 10 min in PBS-T. The blots were rinsed, incubated in Western Blotting Luminol Reagent (Santa Cruz Biotechnologies, CA, USA), and imaged using a Bio-Rad ChemiDoc image analyzer.

### Measurement of Mitochondrial Respiration in Cultured DRG Neurons

An XF24 Analyzer (Seahorse Biosciences, Billerica, MA, USA) was used to measure the neuronal bioenergetic function of rat cultures. The XF24 creates a transient 7-μl chamber in specialized 24-well microplates that allows for the oxygen consumption rate (OCR) to be monitored in real time. Culture medium was changed 1 h before the assay to unbuffered Dulbecco’s modified Eagle’s medium (DMEM, pH 7.4) supplemented with 1 mM pyruvate and 10 mM d-glucose. Neuron density in the range of 2000–4000 cells per well gave a linear OCR. Oligomycin (1 μM), carbonylcyanide-*p*-trifluoromethoxyphenyl hydrazone (FCCP; 1 μM), and rotenone (1 μM) + antimycin A (1 μM) were injected sequentially through ports in the Seahorse FluxPak cartridges. Each loop was started by mixing for 3 min and then delayed for 2 min, and OCR was measured for 3 min. This allowed determination of the basal level of oxygen consumption, the amount of oxygen consumption linked to ATP production, the level of non-ATP–linked oxygen consumption (proton leak), the maximal respiratory capacity, and the non-mitochondrial oxygen consumption. Oligomycin inhibits the ATP synthase leading to a build-up of the proton gradient that inhibits electron flux and determines the coupling efficiency. Injecting FCCP to the culture determines the maximal capacity to reduce oxygen. Finally, rotenone (complex I inhibitor) + antimycin A (complex III inhibitor) was injected to inhibit the flux of electrons and stop the oxygen consumption at cytochrome c oxidase. The remaining OCR revealed after this intervention is primarily non-mitochondrial. Data are expressed as OCR in pmol/min/mg protein.

### Corneal Confocal Microscopy

Type 1 diabetes was induced in female Swiss Webster mice (Envigo, USA) by injection of STZ (Sigma-Aldrich, St Louis, MO, USA) at 6–8 weeks of age in 0.9% saline after overnight fast at 90–100 mg/kg i.p. on two consecutive days, with hyperglycemia (blood glucose > 15 mmol/l confirmed 3 days later in blood obtained by tail prick using a strip-operated reflectance meter) (OneTouch Ultra, LifeScan). CIPN was induced in female Swiss Webster mice by four injections of 5 mg/kg i.p. oxaliplatin given on alternate days for a cumulative dose of 20 mg/kg and confirmed by calculating the 50% paw withdrawal threshold to von Frey filaments, as described in detail elsewhere [56]. Mice were treated with MT7 or vehicle after 6 weeks of diabetes or 4 weeks after the cessation of oxaliplatin treatment. MT7 was dissolved in 0.1 M sodium phosphate buffer to a concentration of 25 ng/ml, and 30 μl of this solution was delivered to the surface of one eye daily, Monday–Friday, for 2 weeks while the contralateral eye received a similar volume of vehicle. Corneal nerves of the sub-basal nerve plexus were imaged in anesthetized mice using a Heidelberg Retina Tomograph 3 with Rostock Cornea Module (Heidelberg Engineering, Heidelberg Germany) as described elsewhere [57]. Corneal nerve density was measured in 5 consecutive images (2-μm intervals) of the sub-basal nerve plexus (SBNP) between the corneal epithelium and stroma and quantified either as occupancy using an 8 × 8 grid layered onto each image [56] or as pixel density following tracing of all visible nerves using ImageJ software [58].

### Statistical Analysis

For each neuronal culture experiment, one rat (age-matched normal or diabetic) was used with cultures performed in replicate and such experiments were independently repeated at least 2–3 times. Data are expressed as mean ± SEM and were subjected to one-way ANOVA with post hoc comparisons using Dunnett’s test for dose-response experiments and for comparison of treatment groups to a single control group. Tukey’s post hoc test was used to determine group means that were significantly different between multiple treatment groups. For in vivo studies, paired or unpaired Student’s *t* tests were performed as defined by the a priori experimental design. GraphPad Prism software was used to perform the statistical analysis.

## Results

### Expression Profile of CaMKs and CaMKKs in Nervous Tissue

**Fig. 1:**
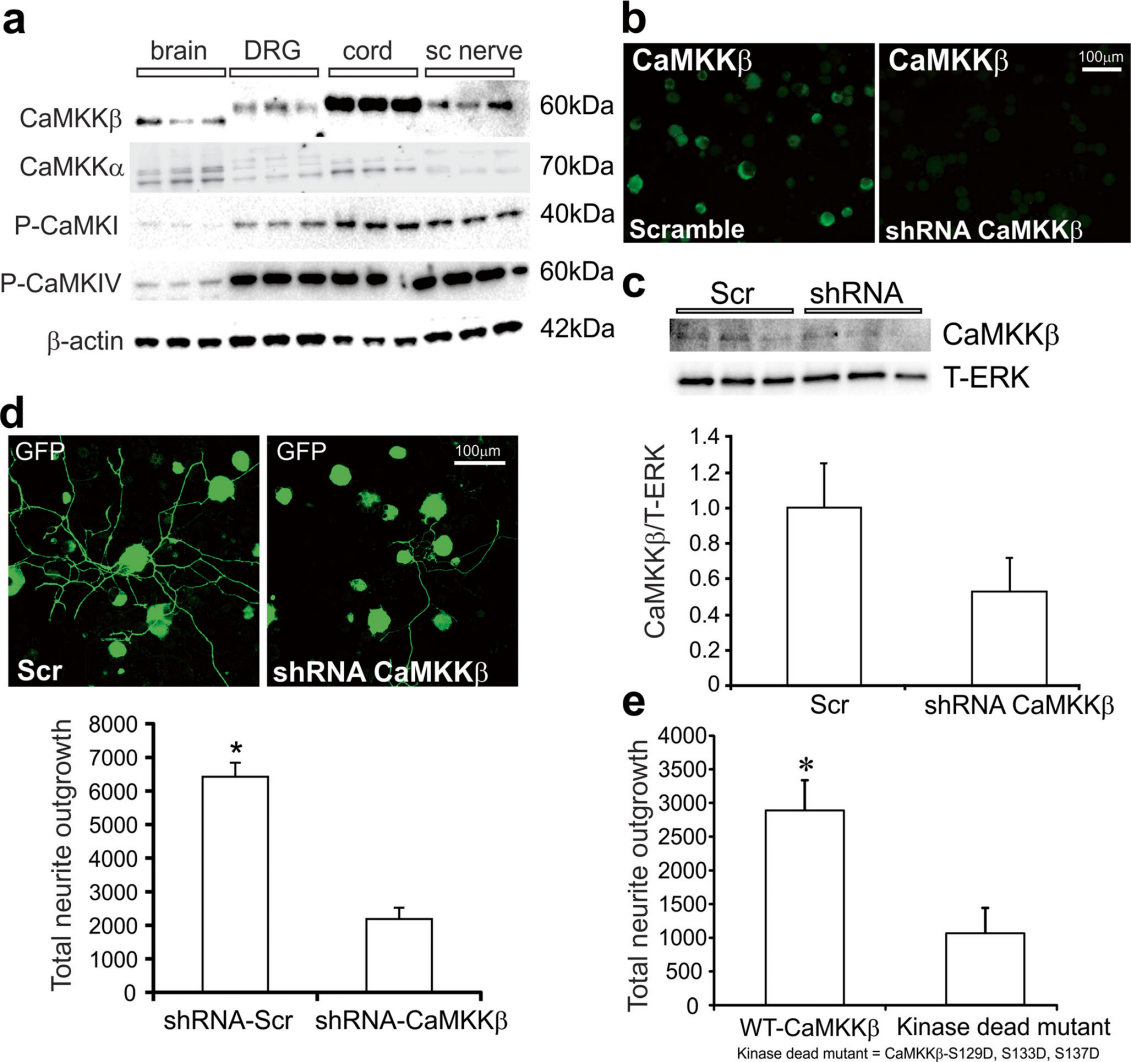
CaMKKβ was expressed by adult sensory neurons and mediated neurite outgrowth. **a** Tissues from an adult normal age-matched rat were analyzed for expression of different forms of CaMK and CaMKKs. sc = nerve sciatic nerve, cord = spinal cord, DRG = lumbar DRG. Each tissue was analyzed in triplicate. **b** Cultured adult sensory neurons were transduced with lentivirus over-expressing scrambled sequence (scr) or shRNA to CaMKKβ for 48 h. Cells were fixed and then immunostained with antibody to CaMKKβ. Bar = 100 μm. **c** In a parallel study, culture lysates were analyzed for CaMKKβ expression using Western blotting and are expressed relative to T-ERK. Values are means ± SEM, *n* = 3 replicate cultures. **d** Effect of shRNA to CaMKKβ on levels of total neurite outgrowth (adjusted for cell number). Constructs co-expressed GFP, and so the GFP fluorescence signal was used for quantification of neurite outgrowth. Values are means ± SEM, *n* = 3 replicate cultures; *p < 0.05 vs shRNA to CaMKKβ by Student’s *t* test. **e** Neurons were transfected for 48 h with GFP-expressing plasmids to wild-type (WT) CaMKKβ or multisite mutant CaMKKβ-S129D, S133D, S137D. Neurite outgrowth is expressed as means ± SEM, *n* = 3 replicate cultures; *p < 0.05 vs mutant by Student’s *t* test

Initially, expression of CaMKKβ in the DRG of adult normal age-matched rats was confirmed. Figure 1a shows that DRG expressed CaMKKβ along with known phosphorylation targets of CaMKKβ, namely CaMK1 and CaMKIV. Interestingly, the relative levels of expression in the DRG and sciatic nerve of CaMKKβ were higher than those seen in the brain. This corresponded with higher levels of phosphorylated CaMK1 and CaMKIV. The CaMKKα isoform was also expressed, although at low levels compared with other tissues.

### CaMKKβ Silencing Restricts Neurite Outgrowth of Adult Sensory Neurons

Dissociated adult sensory neurons derived from a normal adult rat were transduced with lentivirus over-expressing a scrambled sequence or shRNA to CaMKKβ. After 48 h, cultures were fixed and immunostained for CaMKKβ. Figure 1b shows loss of CaMKKβ expression in the presence of shRNA. In a parallel experiment, cell lysates were collected and CaMKKβ protein levels detected by Western blotting. Figure 1c shows an approximately 50% decrease of CaMKKβ in the presence of shRNA. In a supporting experiment, neurons were live-imaged for GFP fluorescence after 48 h of lentiviral transduction and neurite outgrowth was quantified. Figure 1d shows that over-expression of shRNA to CaMKKβ reduced neurite outgrowth in the presence of a cocktail of neurotrophic factors by at least 60% compared with scrambled sequence. A similar inhibition of neurite outgrowth occurred following over-expression of the kinase-dead mutant CaMKKβ-S129D, S133D, S137D (Fig. 1e).

### Pharmacological Blockade of CaMKKβ Inhibits Neurite Outgrowth Driven by M_1_R Antagonists Pirenzepine and MT7

**Fig. 2:**
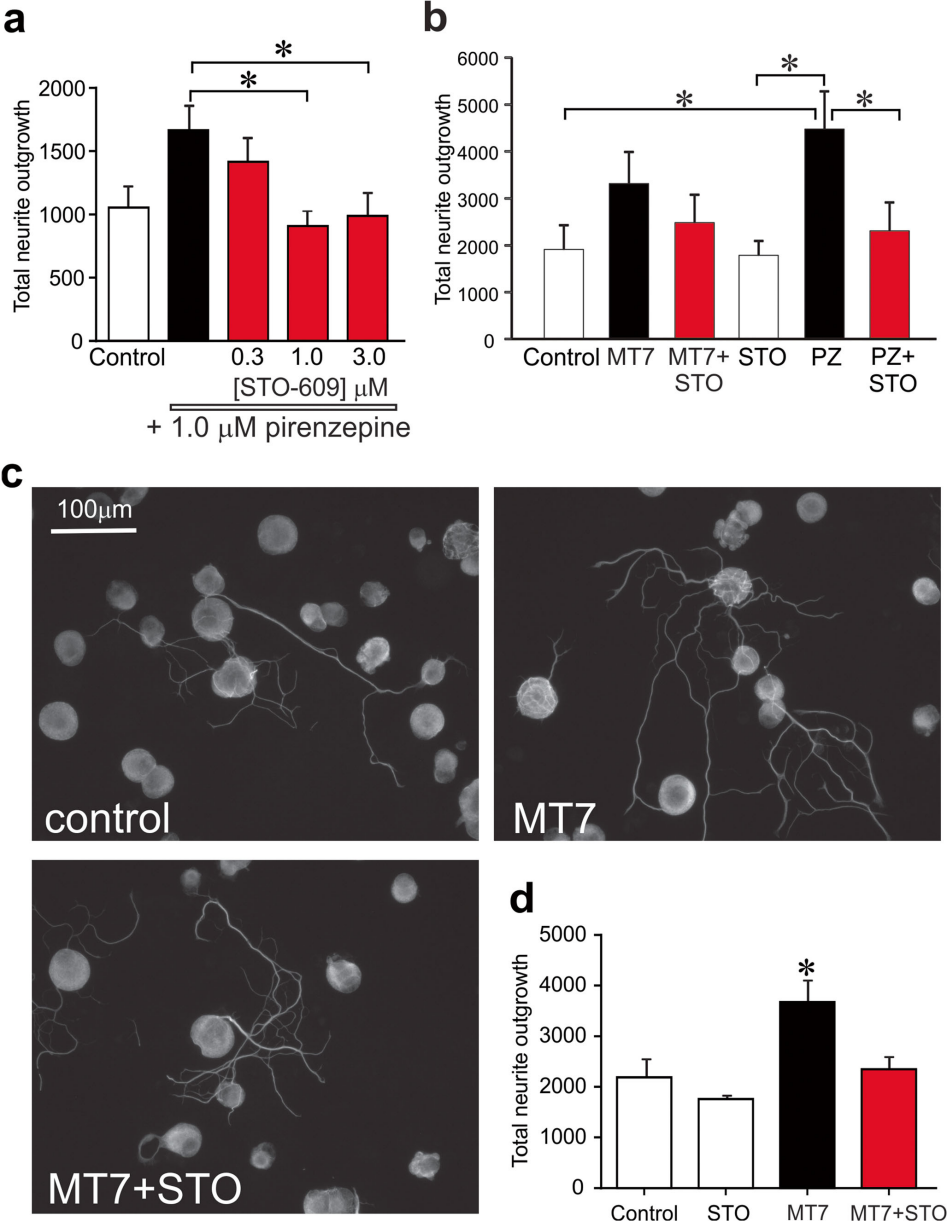
Neurite outgrowth was raised by pirenzepine and MT7 and blocked by the CaMKKβ inhibitor, STO-609. **a** Cultured sensory neurons from a normal rat were exposed to 1 μM pirenzepine and a range of STO-609 concentrations for 24 h (pretreated with STO-609 for 1 h). Cells were fixed and immunostained for β-tubulin III, and neurite outgrowth was assessed. Values are means ± SEM, *n* = 8–10 replicate cultures; *p < 0.05 vs 1.0 μM and 3.0 μM STO-609 by one-way ANOVA with Tukey’s post hoc test. **b** Neurons from a normal rat were cultured for 24 h with 1 μM pirenzepine (PZ) or 100 nM MT7 in the presence of 1 μM STO-609, and neurite outgrowth was analyzed. Values are means ± SEM, *n* = 5–6 replicate cultures; *p < 0.05 vs control, 1.0 μM STO alone, or PZ+STO-609 by one-way ANOVA with Tukey’s post hoc test. **c**, **d** Sensory neurons derived from a 3–5-month STZ-diabetic rat were cultured for 24 h in the presence of 100 nM MT7 with/without 1 μM STO-609. Fluorescent images were taken from cells immunostained for β-tubulin III; bar = 100 μm. In **d**, bar charts of data where values are means ± SEM, *n* = 6 replicate cultures; *p < 0.05 vs other groups by one-way ANOVA with Tukey’s post hoc test

In a previous study, we demonstrated that selective or specific M_1_R antagonists increased neurite outgrowth in adult sensory neurons [42]. In Fig. 2a, treatment for 24 h with the selective M_1_R antagonist, pirenzepine at 1.0 μM, increased neurite outgrowth of normal adult sensory neurons by approximately 50% and this effect was dose-dependently blocked by the CaMKK inhibitor, STO-609. In a follow-up study, 1 μM pirenzepine elevated neurite outgrowth by approximately 50–60% and the CaMKKβ inhibitor, STO-609, completely blocked neurite outgrowth at 1 μM (Fig. 2b) whereas STO-609 alone had no effect on background neurite outgrowth supported by the cocktail of neurotrophic factors. To confirm this neuritogenic pathway was relevant in the setting of diabetic neuropathy, sensory neuron cultures were prepared from a 3–5-month-old STZ-diabetic rat and exposed to the specific M_1_R antagonist MT7 at 100 nM, with/without 1 μM STO-609 for 24 h. Figure 2c presents representative images of cultures immunostained for neuron-specific β-tubulin III. MT7 induced an approximately 40% elevation in neurite outgrowth that was completely blocked by STO-609 (Fig. 2d), replicating the pattern seen in the DRG from normal rats (Fig. 2b).

### MT7 Activated AMPK in a Dose and Time–Dependent Manner and Was Mediated Via CaMKKβ

**Fig. 3:**
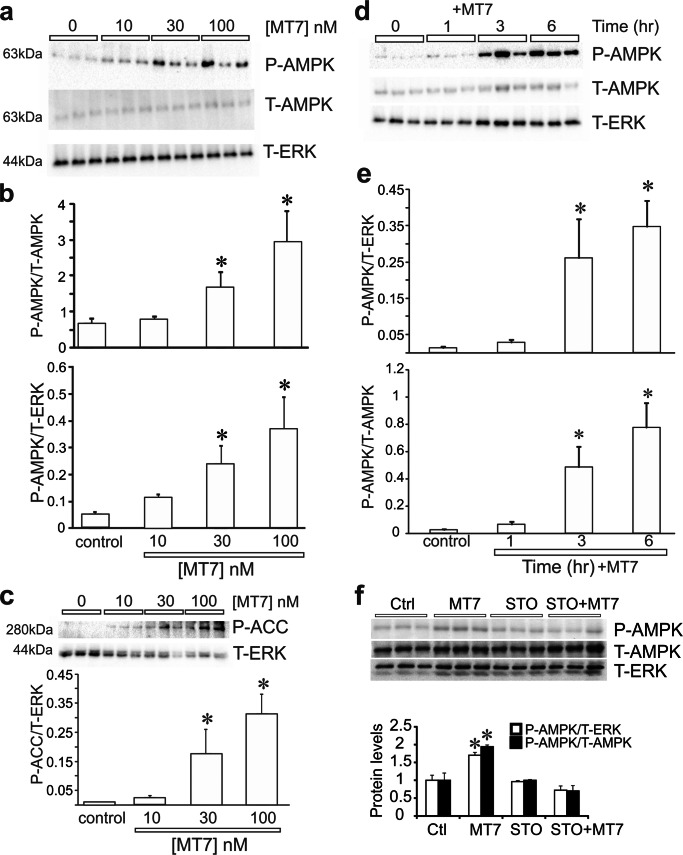
MT7 elevated phosphorylation of AMPK in a dose and time–dependent manner. **a** Sensory neurons derived from a 3–5-month STZ-diabetic rat were cultured overnight and then treated with varying doses of MT7 for 1 h. Western blots are shown for P-AMPK, T-AMPK, and T-ERK. **b** Levels of expression of these proteins presented relative to T-ERK or T-AMPK. **c** Samples from the experiment in **b** were probed for P-ACC. **d** Neurons from a diabetic rat were cultured overnight and then exposed to 100 nM MT7 for varying times. **e** Levels of P-AMPK presented relative to T-ERK and T-AMPK. For **b**, **c**, and **e**, values are means ± SEM, *n* = 3 replicate cultures; *p < 0.05 vs control by one-way ANOVA with Dunnett’s post hoc test. **f** Pharmacological blockade of CaMKKβ prevents MT7-dependent phosphorylation of AMPK. Neurons from a control rat were cultured overnight and then pretreated with 1 μM STO-609 for 2 h and then treated with 100 nM MT7 for 3 h. Levels of P-AMPK presented relative to T-ERK and T-AMPK. Values are means ± SEM, *n* = 3 replicate cultures; *p < 0.05 vs other groups by one-way ANOVA with Tukey’s post hoc test

**Fig. 4:**
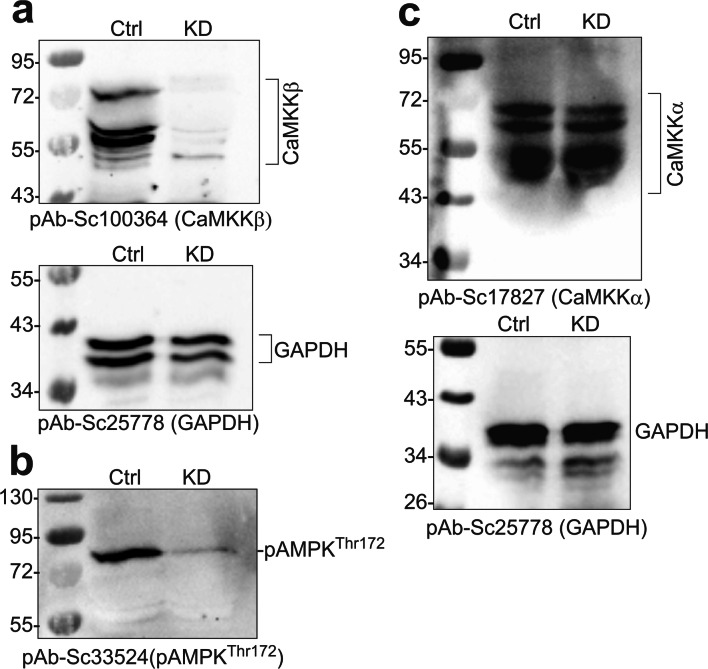
Knockdown of CaMKKβ reduces AMPK phosphorylation. **a**–**c** Immunoblots showing the relative expression of **a** CaMKKβ, **b** pAMPK (on Thr-172), and **c** CaMKKα in control (scrambled siRNA) and CaMKKβ siRNA–transfected cultured adult rat primary DRG neurons (GAPDH used as a loading control in **a** and **c** from normal rats). Adult rat DRGs were freshly dissected, dissociated, and transfected with siRNAs using Amaxa nucleofection reagent and cultured in defined media for 48 h. The total proteins were separated by SDS-PAGE and immunoblotted

CaMKKβ is an endogenous activator of AMPK [22, 23], so we determined if MT7 could activate AMPK in cultures derived from STZ-induced diabetic rats. In Fig. 3a, Western blots reveal a marked dose-dependent elevation in phosphorylation of AMPK following 1 h of MT7 treatment. Quantification of P-AMPK relative to T-AMPK or T-ERK revealed an at least 5-fold elevation at 100 nM MT7 (Fig. 3b). The endogenous target of activated AMPK, P-ACC, was also significantly enhanced (Fig. 3c). A time course for the effect of 100 nM MT7 was performed and revealed a remarkable elevation in P-AMPK levels following 6 h of treatment (Fig. 3d, e). At 1 h, 100 nM MT7 caused a 2-fold elevation in P-AMPK, although this was not statistically significant and not as robust as compared with the data revealed in Fig. 3a. To confirm the MT7-dependent phosphorylation was mediated via CaMKKβ, neuronal cultures were treated with 100 nM MT7 for 3 h in the presence/absence of 1 μM STO-609. The inhibitor completely blocked the MT7 induction of P-AMPK levels (Fig. 3f). To confirm that CaMKKβ was regulating AMPK phosphorylation status, we used lipid nanoparticles to deliver a cocktail of siRNAs to CaMKKβ to normal DRG cultures. This approach gives a superior level of knockdown for molecular endpoints, such as gene expression analysis, compared with the shRNA technique, since 80–90% of neurons were affected. Following 48 h of treatment with the siRNAs, the level of CaMKKβ protein was clearly depleted (Fig. 4a). This was a specific knockdown since the related CaMKKα protein was not affected (Fig. 4c). Using standard Western blotting, the knockdown of CaMKKβ also caused a reduction of the P-AMPK signal (Fig. 4b).

### MT7 Elevated Transcriptional Activity of the AMPK target, PGC-1α, Via CaMKKβ

**Fig. 5:**
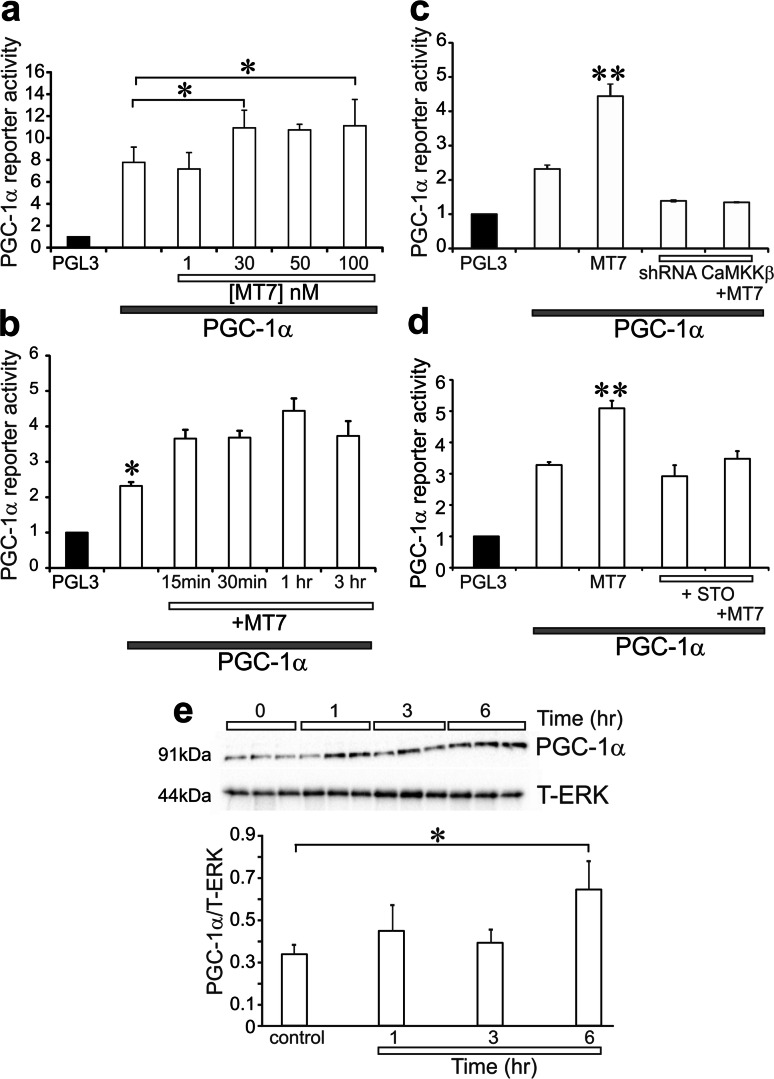
MT7 enhanced transcriptional activity of PGC-1α through a CaMKKβ-dependent pathway. **a** Neurons from a diabetic rat were transfected with reporter plasmid for PGC-1α and, then after 48 h, were treated with varying doses of MT7 for 1 h. Cell lysates underwent dual-luciferase reporter assay for PGC-1α and data presented relative to PGL3 internal control plasmid (indicated by black bar). Values are means ± SEM, *n* = 3 replicate cultures; *p < 0.05 vs no treatment by one-way ANOVA with Tukey’s post hoc test. **b** Time course for the effect of 100 nM MT7 on PGC-1α transcriptional activity. Values are means ± SEM, *n* = 3 replicate cultures; *p < 0.05 vs all time points by one-way ANOVA with Tukey’s post hoc test. **c** Neurons from a diabetic rat were transfected with the PGC-1α reporter plasmid and, then after 48 h, were transduced with lentivirus over-expressing shRNA to CaMKKβ. After 48 h, cultures were treated with 100 nM MT7 for 1 h and the reporter activity was determined. Values are means ± SEM, *n* = 3 replicate cultures; **p < 0.01 vs other groups by one-way ANOVA with Tukey’s post hoc test. **d** Neurons from a diabetic rat were transfected with the PGC-1α plasmid for 48 h. After this period, neurons were then pretreated with 1 μM STO-609 for 2 h and then treated with 100 nM MT7 for 1 h and PGC-1α reporter activity was determined. Values are means ± SEM, *n* = 3 replicate cultures; **p < 0.01 vs other groups by one-way ANOVA with Tukey’s post hoc test. **e** Diabetic neurons were cultured overnight and then exposed to 100 nM MT7 for varying periods of time. A crude nuclear sample was collected and subjected to Western blotting for PGC-1α. Values presented relative to T-ERK and are means ± SEM, *n* = 3 replicate cultures; *p < 0.05 vs control by one-way ANOVA with Dunnett’s post hoc test

The pathway downstream from activation of AMPK that mediates mitochondrial biogenesis and refurbishment includes activation of PGC-1α leading to enhancement of its cotranscriptional activities [25, 26]. Cultures derived from STZ-diabetic rats were exposed to varying doses of MT7 for 30 min and cell lysates analyzed using a dual-luciferase assay for PGC-1α transcriptional activity. Figure 5a shows that 30–100 nM MT7 significantly elevated PGC-1α reporter activity. Figure 5b demonstrates that 100 nM MT7 significantly raised PGC-1α transcriptional activity as early as 15 min and this was sustained for up to 3 h. We used two approaches to causally link MT7 activation of PGC-1α transcriptional activity to upstream signaling via CaMKKβ. Neuronal cultures from STZ-diabetic rats were cotreated with either 100 nM MT7 and shRNA to CaMKKβ (Fig. 5c) or 1 μM STO-609 (Fig. 5d). Under both conditions of CaMKKβ inhibition, the ability of MT7 to enhance PGC-1α transcriptional activity was abolished. Finally, in support of the dual-luciferase reporter approach, we used Western blotting to determine levels of PGC-1α protein in lysates from neuronal cultures derived from STZ-diabetic rats. Treatment with 100 nM MT7 for 6 h significantly increased PGC-1α protein levels (Fig. 5e).

### MT7 Augmented Mitochondrial Function Through a CaMKKβ-Dependent Pathway

**Fig. 6:**
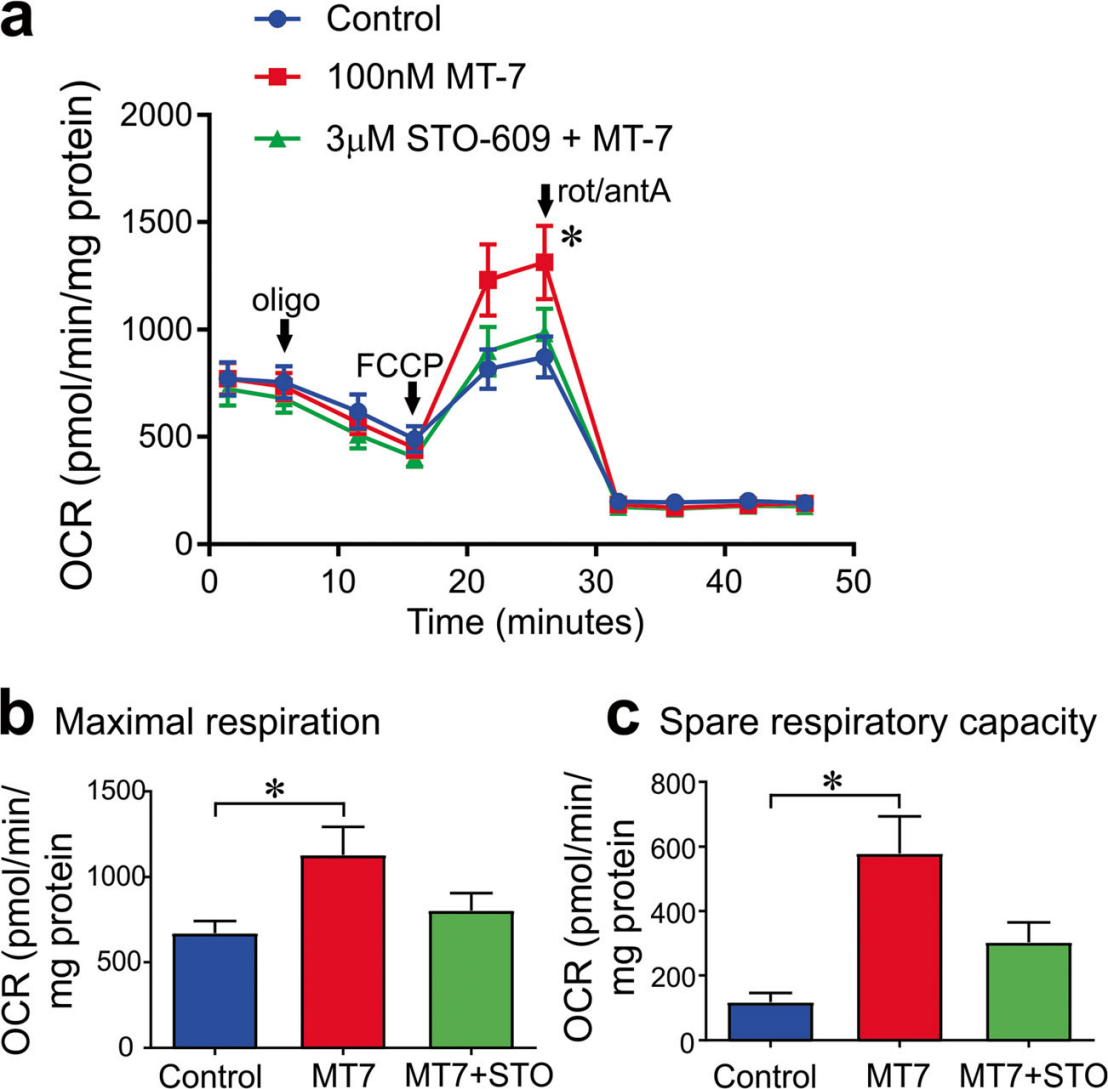
MT7 improves mitochondrial function in sensory neurons cultured from STZ-induced diabetic rats. **a** Oxygen consumption rate (OCR) was measured in the DRG from a 6-week-old diabetic rat cultured overnight and in the presence or absence of 100 nM MT7 for 1 h and in the presence or absence of a 3-h STO-609 (3 μM) pretreatment. OCR in pmol/min was normalized to mg of protein. **b** Maximal respiration and **c** spare respiratory capacity were determined after subtracting the non-mitochondrial OCR. Values are mean ± SEM, *n* = 6–7 replicate cultures, and adjusted to total protein levels. *p < 0.05 control vs MT-7 (one-way ANOVA with Tukey’s post hoc test)

Neuronal cultures derived from STZ-diabetic rats were treated for 3 h with 100 nM MT7 ± STO-609 followed by assessment of bioenergetic profile using the Seahorse XF24 Analyzer. Maximal respiration and spare respiratory capacity were significantly increased by MT7, and this effect was blocked by STO-609 (Fig. 6; basal respiration and the coupling efficiency were unaffected by MT7 treatment).

### Topical MT7 Reverses Corneal Fiber Loss in STZ-Diabetic and CIPN Mice

**Fig. 7:**
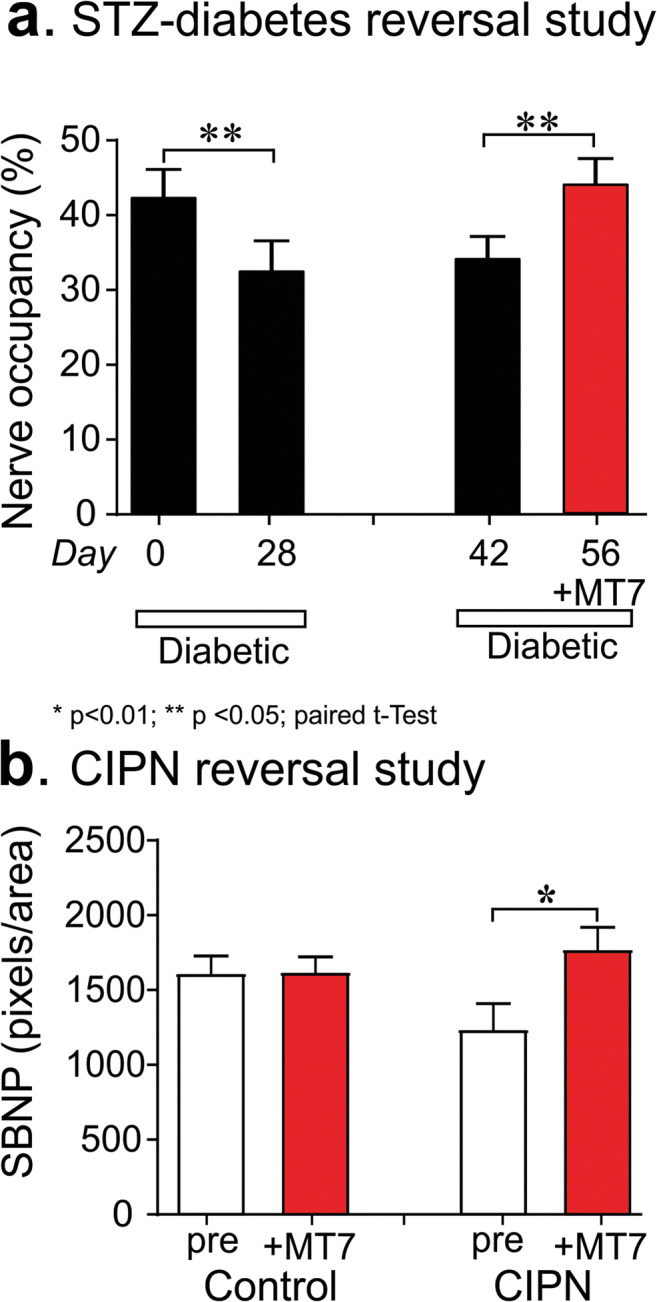
Topical MT7 promotes increased corneal nerve density in diabetic and CIPN mice. **a** Corneal nerve density measured by confocal microscopy in female Swiss Webster mice before induction of diabetes (day 0) and 28 days later. The same mice underwent further corneal nerve measurements after 42 days of diabetes before receiving daily delivery of MT7 to one eye (30 μl of 25 ng/ml solution), Monday–Friday, for 2 weeks. Sub-basal nerve plexus (SBNP) occupancy was calculated by tracing nerves and using a grid overlay system [56]. **b** Corneal nerve density (SBNP) was measured by confocal microscopy in control or oxaliplatin-treated (CIPN) female Swiss Webster mice before (pre) and 2 weeks after (+MT7) daily delivery of MT7 to one eye (30 μl of 25 ng/ml solution), Monday–Friday, for 2 weeks. In this study, the SBNP was measured by tracing all nerves and recording total pixels/image area [58]. Data are group mean + SEM of *N* = 7–9/group. Within-group statistical comparisons before and after treatment with STZ or MT7 by paired Student’s *t* test; *p < 0.05, **p < 0.01

**Fig. 8:**
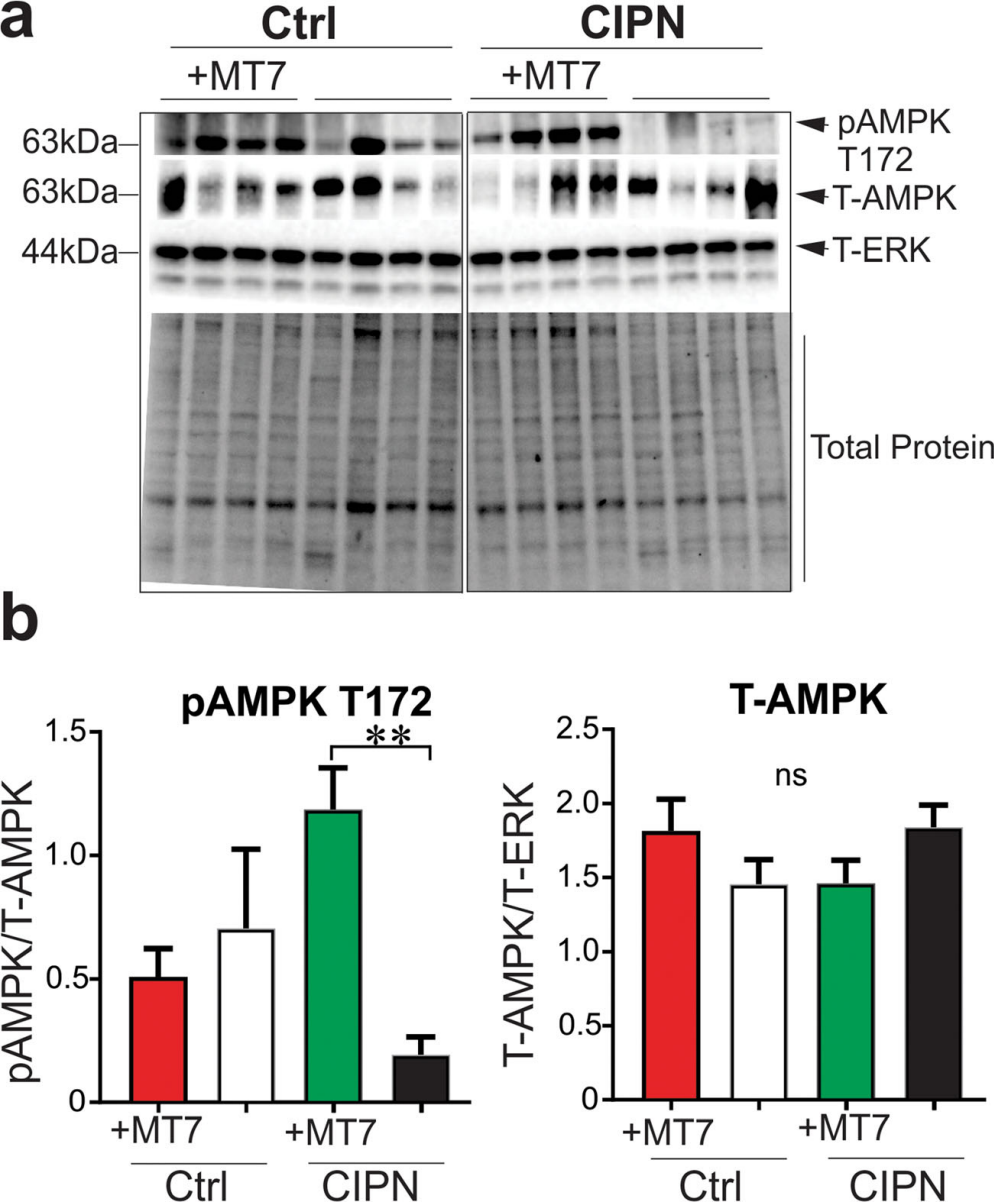
Topical MT7 to the eye restored AMPK phosphorylation in the ipsilateral trigeminal ganglia (TG) in CIPN mice (oxaliplatin-treated; CIPN). Control or CIPN mice were treated topically to the left eye (ipsilateral) with MT7 using a stock of 25 ng/ml, and 30 μl of this solution was delivered to the surface of one eye daily, Monday–Friday, for 2 weeks. The right (contralateral) eye received vehicle. At the end of the study, the ipsilateral and contralateral TG from control (Ctrl) or CIPN mice were isolated and subjected to **a** Western blotting. **b** Western blot band intensities of phosphorylated AMPK (P-AMPK) and total AMPK (T-AMPK) were normalized to T-AMPK or T-ERK, respectively. The +MT7 is the treated (ipsilateral) eye, and without MT7 reflects the untreated (contralateral) eye. Data are mean ± SEM of *N* = 4 (ganglia were combined from two mice; 8 mice dissected to give *n* = 4); **p < 0.01 by paired Student’s *t* test

Nerve density in the corneal sub-basal plexus was measured by corneal confocal microscopy before and 4 weeks after induction of diabetes. Four weeks of diabetes caused a significant (*p* < 0.05, paired Student’s *t* test) reduction in corneal nerve density (measured as occupancy) that also persisted at week 6 of diabetes (Fig. 7a). Topical delivery of MT-7 for 10 days significantly (*p* < 0.05, paired Student’s *t* test) increased corneal nerve density to values similar to those before the onset of diabetes. In a separate study, corneal nerve density (measured as pixels per unit area) was quantified in mice that had been treated with vehicle (control) or oxaliplatin 3 weeks earlier. Oxaliplatin-injected mice exhibited CIPN, as illustrated by the presence of increased sensitivity to the touch of von Frey filaments (50% paw withdrawal threshold for vehicle group = 1.50 ± 0.1 vs CIPN group = 0.76 ± 0.14 g: *p* < 0.01 by unpaired Student’s *t* test, *N* = 8–9/group) and tended towards lower corneal nerve density than control mice (Fig. 7b). At this time, both control and CIPN mice were treated topically with MT7 to one eye for 2 weeks. MT7 treatment significantly increased corneal nerve density of CIPN mice compared to pre-MT7 treatment values (*p* < 0.01 by paired Student’s *t* test: Fig. 7b) but was without significant effect on nerve fiber density in control mice. At the end of the study, the ipsilateral and contralateral trigeminal ganglia were removed and subjected to Western blotting to quantify P-AMPK levels. Figure 8 shows that, in oxaliplatin-treated mice (CIPN), MT7 caused a significant elevation of P-AMPK levels in the ipsilateral trigeminal ganglion when compared to the contralateral trigeminal ganglion (corresponding to the vehicle-treated eye), when adjusted for T-AMPK (similar results were obtained when adjusted to T-ERK or total protein; data not shown). Also, note that P-AMPK levels were reduced in the trigeminal ganglia of the untreated side in CIPN mice vs control mice (although this effect did not reach statistical significance). MT7 treatment to one eye of normal mice did not result in significant differences of P-AMPK levels between the ipsilateral and contralateral trigeminal ganglia.

## Discussion

Our previous work identified the AMPK/PGC-1α signaling cassette as an important target for the ability of M_1_R antagonists to enhance mitochondrial activity and promote neurite outgrowth from adult sensory neurons and to protect animal models against peripheral neuropathy [42]. The present study demonstrates for the first time that blockade of the M_1_R by the specific antagonist MT7 drives axonal outgrowth via CaMKKβ and subsequent activation of the AMPK/PGC-1α axis to augment mitochondrial function. Our in vitro assay for neurite outgrowth of DRG neurons is a useful model of axonal plasticity in vivo since it replicates the process of collateral sprouting [50]. Thus, this assay can be a predictor of efficacy with respect to drug effects on IENF density (see [42]) and fiber levels in the cornea. In this regard, topical MT7 also reversed loss of sensory nerves in the cornea of mice with neuropathy caused by diabetes or oxaliplatin-induced CIPN. Interestingly, the topical application of MT7 to one eye of CIPN mice enhanced AMPK activation only in the ipsilateral trigeminal ganglion, suggesting the importance of the interaction of MT7 with distal peripheral regions of sensory nerve fibers.

This is the first demonstration of a role for CaMKKβ in directing neurite outgrowth in adult peripheral sensory neurons. Previous studies in embryonic CNS neurons have shown that CaMKKβ is implicated in a variety of pathways driving neurite outgrowth and axonal plasticity. The phosphorylation state of CaMKKβ also impacts the formation of neurites and polarity during differentiation [51]. Knockout or pharmacological blockade of CaMKKβ in embryonic cerebellar granule cells [51], hippocampal neurons [59], and neuroblastoma cells [60] resulted in inhibition of neurite outgrowth, with CaMKKβ localized to neurites and growth cones [51, 60]. Activation of CaMKI, a primary downstream target of CaMKKβ, has also been mechanistically linked to the development of axonal projections in both embryonic cortical and hippocampal neurons [61]. Our studies extend this work to the PNS and place CaMKKβ downstream of the M_1_R and upstream of AMPK and PGC-1α in a signaling cascade that drives neurite outgrowth from adult sensory neurons.

Other than driving neurite growth, activation of the CaMKKβ/AMPK signaling pathway has also been implicated in protection from neurological impairments such as ischemic stroke and hypoxic-ischemic encephalopathy [62, 63]. A potentially key finding in understanding this neuroprotective property is that CaMKKβ lies upstream from AMPK to augment mitochondrial function. Previous studies have highlighted that activation of AMPK can elevate neurite outgrowth. For example, activation of AMPK by resveratrol elevates neurite outgrowth in embryonic sensory neurons [64]. Interestingly, this resveratrol-triggered pathway to AMPK activation in sensory neurons was independent of CaMKK [64], although in cortical neurons, CaMKK was activated upstream from AMPK, demonstrating the importance of cellular context when considering these signaling cassettes. Our previous work in adult sensory neurons showed that resveratrol-driven neurite outgrowth could be blocked with adenovirus-mediated delivery of dominant negative mutants of AMPK [29]. Furthermore, in STZ-induced diabetic rats, resveratrol therapy activated AMPK and mitochondrial function in the DRG and protected from sensory neuropathy [29]. In adult sensory neurons, M_1_R blockade also augments mitochondrial function via AMPK and protects from sensory neuropathy [42]. In the present study, MT7 elevated AMPK phosphorylation by at least 2–3-fold within 1 h and, at longer time points, caused a very robust elevation of P-AMPK that exceeded levels previously seen with the classical AMPK activator, resveratrol. In contrast, acute treatment with MT7 for up to 1 h caused no significant activation of AMPK, suggesting that there are several intermediate steps upstream of AMPK, presumably proximal to CaMKKβ, that are mediated by antagonism of the M_1_R (discussed below).

In this study, the majority of the work was performed with DRG cultures derived from diabetic rats. The rationale for this approach was that we have found responses to antimuscarinic drugs to be far more robust in diabetic cultures, particularly with respect to activation of AMPK and augmentation of mitochondrial function. Previous in vitro and in vivo studies with DRG neurons have revealed that the type 1 or type 2 diabetic state triggers downregulation of AMPK [29, 65]. This hyperglycemia-induced downregulation of AMPK and PGC-1α is also observed in the skeletal muscle, liver, kidney, and cardiac tissue [29, 66–72]. Therefore, we surmise that this suppression of the key energy sensor in the neuron thus engenders greater sensitivity to putative stimulators of AMPK and mitochondrial function.

PGC-1α is a key mediator of AMPK activation to enhance mitochondrial function, and activation of PGC-1α is neuroprotective in animal models of Huntington and Alzheimer’s diseases [73–75]. Further, impaired PGC-1α signaling has recently been implicated in Parkinson’s disease [75, 76] and causes CNS lesions linked to impaired neurite outgrowth and hyperactivity [77, 78]. PGC-1α was activated within 1 h by MT7 treatment in a CaMKKβ-dependent fashion. There was significant activation of PGC-1α transcriptional activity by 15 min which did precede evidence of any AMPK activation. Thus, it is feasible that other MT7 triggered pathways, independent of CaMKKβ, may be contributing to PGC-1α activation.

A potential therapeutic application of the consequences of M_1_R antagonism by MT7 is illustrated by our findings that MT7 delivered topically to the eye was effective in reversing reduced corneal nerve density caused by diabetes or CIPN. This, along with our previous finding that topical MT7 also prevented and reversed corneal nerve loss induced by the neurotoxic HIV-associated protein gp120 [42], supports the concept that M_1_R antagonism promotes peripheral nerve regenerative growth irrespective of the primary pathogenesis of the underlying neuropathy. Reduced corneal nerve density also occurs in patients with diabetic neuropathy [79] or cancer patients treated with oxaliplatin [80] and is emerging as an early biomarker for a diverse collection of neurodegenerative diseases in the PNS and CNS [81]. The promotion of corneal nerve regrowth in disease models by topical application of antimuscarinics may therefore serve as a predictor of their broader efficacy in peripheral nerves. Indeed, in the case of diabetic neuropathy, systemic delivery of the M_1_R-selective antagonist pirenzepine both prevents and reverses multiple functional and structural indices of peripheral neuropathy in diabetic rodents [42]. It is pertinent to note that MT7 was also very effective in activating AMPK in the trigeminal ganglia of CIPN mice, but only on the ipsilateral side. Access of MT7 to the cell bodies of the trigeminal ganglion is unlikely to be via a systemic route, otherwise the contralateral trigeminal would also have shown elevated AMPK activity. This supports the idea that specific M_1_R blockade, and subsequent activation of AMPK within the cell body, is mediated via an initial signal derived from the nerve ending in the cornea. This has important implications in terms of a future therapeutic approach in humans with peripheral neuropathy as topical delivery of M_1_R antagonists to sensory nerve endings may prove sufficient to activate AMPK in neuronal cell bodies and promote neuroprotection and nerve regrowth without requiring concurrent systemic exposure at concentrations likely to block M_1_R in other organs.

The mechanism by which M_1_R antagonism leads to activation of CaMKKβ and subsequent mobilization of the AMPK/PGC-1α signaling axis can only be speculative at this juncture. Key requirements for CaMKKβ activation were a rise in intracellular Ca^2+^ and corresponding availability of calmodulin [27]. It is unlikely this source of Ca^2+^ is intracellular, for example from the endoplasmic reticulum (ER), since M_1_R antagonism blocks ACh-induced ER Ca^2+^ release driven by IP_3_, which itself is generated via G protein/PLC-dependent PIP_2_ breakdown. Many Ca^2+^ channels interact with PIP_2_ at the membrane, including diverse TRP channels [82] many of which are non-selective cation channels [83]. Interestingly, TRPM3 is one of the few TRP-type cation channels that is closed under low PIP_2_ levels—as observed in the presence of ACh-induced IP_3_ generation [84, 85]. Thus, antagonism of the M_1_R could lead to opening of TRPM3 and a local rise in [Ca^2+^]. TRPM3 is expressed in human and rodent DRG sensory neurons with a function linked to thermal sensory perception [83, 86, 87]. TRPM3 also has an intracellular binding site for Ca^2+^ and calmodulin that could act as a scaffold for CaMKKβ activation [88, 89]. Whether TRPM3 serves as a Ca^2+^ modulator following M_1_R antagonism by MT7 is currently under investigation. Another possible mediator of cytoplasmic Ca^2+^ following M_1_R blockade is activation of STIM2, a Ca^2+^ sensor that can trigger CaMKKβ and AMPK [90].

## Conclusions

We have demonstrated that CaMKKβ is a key signaling molecule that mediates neurite outgrowth and drives axonal repair in neurodegenerative diseases of the peripheral nervous system. This enzyme lies upstream from the critical energy sensing pathway comprising AMPK/PGC-1α which regulates mitochondrial function to enhance axonal growth and protect from neurodegeneration. The ability of a topically applied antimuscarinic drug to activate this pathway in vivo suggests novel approaches to promote nerve regeneration in diseases of the PNS.

## Data Availability

The datasets used and/or analyzed during the current study are available from the corresponding author on reasonable request.
